# Allogeneic hematopoietic stem cell transplant for familial hemophagocytic lymphohistiocytosis: a case report and literature review

**DOI:** 10.3389/fimmu.2024.1391074

**Published:** 2024-06-03

**Authors:** Liu Bingjie, Zhang Linlin, Ma Futian, Xuan Fan, Du Huan, Xiaoli Wu, Lixia Zhou, Wang Fuxu, Zhang Xuejun, Wang Ying

**Affiliations:** ^1^ Department of Hematology & Hematology Institute, The Second Hospital of Hebei Medical University, Shijiazhuang, Hebei, China; ^2^ Department of Pediatric Hematology, The Second Hospital of Hebei Medical University, Shijiazhuang, Hebei, China; ^3^ Department of Imaging, The Second Hospital of Hebei Medical University, Shijiazhuang, Hebei, China

**Keywords:** cytokine storm, white matter damage, hematopoietic stem cell transplant (HSCT), UNC13D gene, familial hemophagocytic lymphohistiocytosis (FHL)

## Abstract

**Objectives:**

This study aims to discuss the clinical manifestations and treatment of Familial hemophagocytic lymphohistiocytosis (FHL) caused by a mutation in the UNC13D gene.

**Methods:**

A 6-year-old female child presented with unexplained febricity, splenomegaly, pancytopenia, hemophagocytic lymphohistiocytosis in bone marrow, decreased NK cell activity, soluble CD25 levels > 44000ng/ml. Genetic sequencing revealed a mutation in the UNC13D gene. Additionally, the patient experienced intermittent fever with seizures characterized by involuntary twitching of the left upper limb. Head magnetic resonance imaging (MRI) showed white matter lesions.

**Results:**

According to the HLH-2004 diagnostic criteria revised by the International Society of Histiocytosis the patient was diagnosed with FHL. Despite receiving HLH-2004 treatment, the disease relapsed. However, after a salvage allogeneic Hematopoietic Stem Cell Transplant (HSCT), febricity, abnormal blood cells, and neurological symptoms significantly improved.

**Conclusions:**

Prompt performance of allogeneic HSCT is crucial upon diagnosis of FHL, especially when neurological involvement is present.

## 1 Introduction

Familial hemophagocytic lymphohistiocytosis (FHL) represents a critical congenital immunodeficiency, stemming from genetic anomalies that disrupt the cytotoxic functions of T cells and natural killer (NK) cells (1). The clinical symptoms of patients with FHL may vary widely. Patients with FHL may present with a variety of neurological symptoms prior to or after diagnosis, which in some cases can be fatal (2). Recent advancements in treatment have significantly improved the prognosis for FHL patients, offering potential cures through immunotherapy and hematopoietic stem cell transplantation (HSCT) (3). Nonspecific symptoms in the early phase may delay the diagnosis of FHL. The initial nonspecific symptoms of FHL can lead to diagnostic delays. Reports have surfaced of FHL cases involving cerebellar swelling and subsequent obstructive hydrocephalus (4, 5). However, the lack of distinctive clinical indicators often results in misdiagnosis or under-diagnosis within medical settings. This study presents a case of FHL-3 with neurological symptoms. After a 3-year delay in diagnosis, a FHL-3 patient underwent successful salvage allogeneic HSCT, restoring her normal hematopoietic function and saving her life. She has remained disease-free for 4 years. The report will discuss its clinical features, diagnostic process, treatment strategies, and synthesizes pertinent literature in this field.

## 2 Case information

### 2.1 Diagnosis and chemotherapy

**Figure 1:**
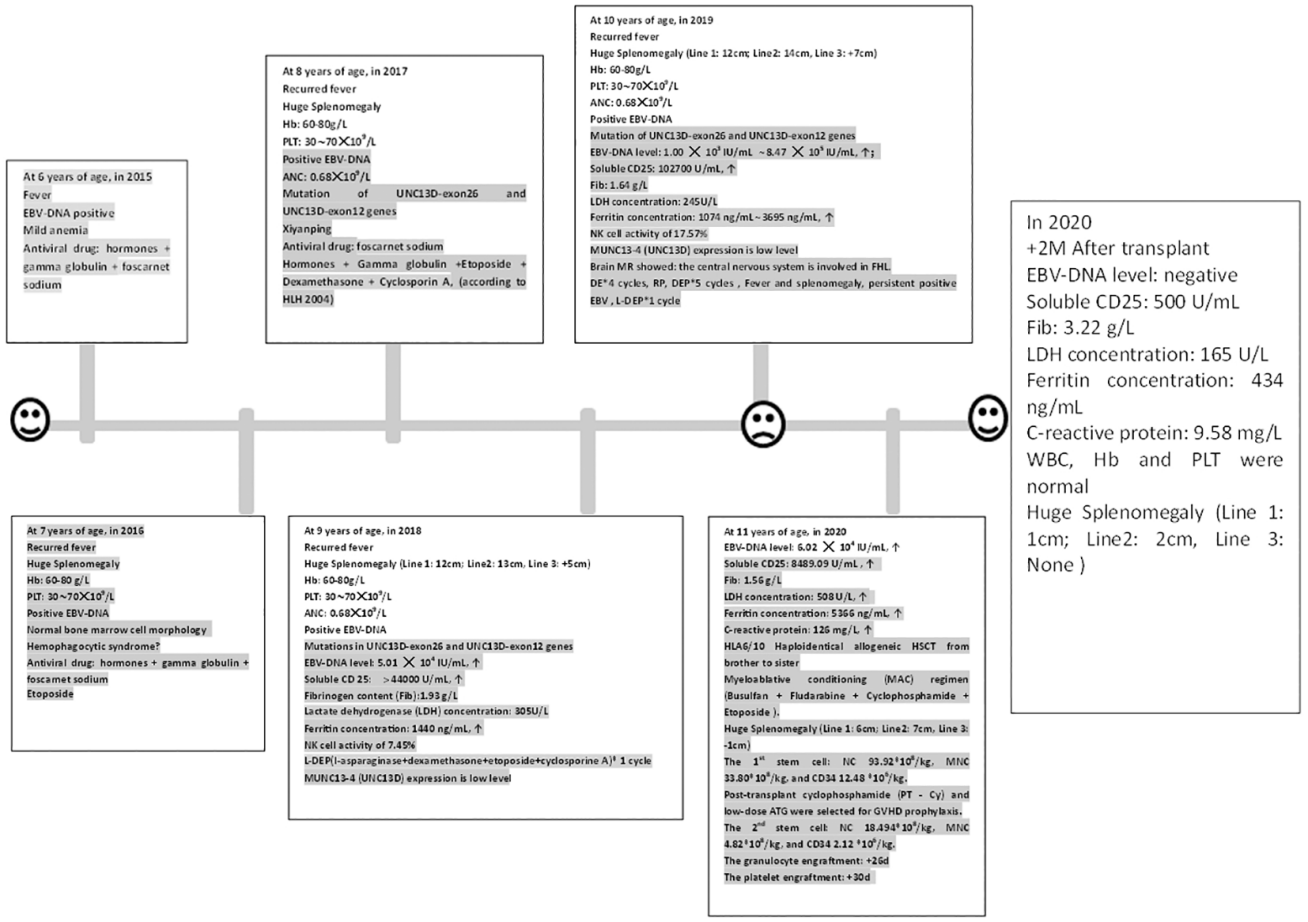
The clinical course and peripheral blood counts of the case. The horizontal axis shows the time course of this case, with one scale representing 1 year. Hb, Hemoglobin; Plt, Platelet count; ANC, Absolute neutrophil count; ↑: Beyond the normal upper limit. RP: The patient got complete remission after DE 2 cycles; but she was relapsed after the fourth cycle.

The clinical course of our case is shown in **Figure 1**. A 6-year-old female patient, weighing 13 kg, presented with a fever of 38.6°C in November 2014. The initial examination revealed no palpable liver or spleen enlargement. The relevant examination results showed EBV-DNA positive and mild anemia. After accepting antiviral drugs (foscarnet sodium and sodium chloride, 40mg/Kg/d, d1-d14), steroid (dexamethasone, 10mg/M^2^, 7.5mg/m^2^, according to the HLH-2004 protocol) and gamma globulin (human immunoglobulin (pH4) for intravenous injection, 5g/d, 1/10d). Subsequently, the patient’s condition improved. However, in February 2015, she experienced a recurrence of fever, now with splenomegaly and moderate anemia, and a new finding of thrombocytopenia with platelet counts oscillating between 60 and 70 × 10^9^/L. Despite persistent EBV-DNA positivity and normal bone marrow cell morphology, a definitive diagnosis remained elusive. After receiving steroid treatment the spleen shrunk and the results of blood routine test returned to normal. When the fever recurred in December 2015, the physical examination still showed splenomegaly (located at the sub-umbilical level), and the blood routine test showed a moderate anemia (60∼80g/L) and a thrombocytopenia (30∼70 × 10^9^/L), with continuously positive EBV-DNA. The bone marrow cell morphology was still normal. Ferritin, triglycerides and fibrinogen were all within the normal range. Considering the possibility of hemophagocytic syndrome, the patient’s condition improved after receiving two doses of etoposide and 8-day dexamethasone and cyclosporin A treatments (according to HLH2004). In November 2017, the patient exhibited similar clinical signs. The gene mutation analysis report showed mutations in UNC13D-exon26 and UNC13D-exon12 genes, with the specific mutation sites of c.1055 + 1G > A and c.2448–13G>A. After receiving intravenous Xiyanping (a traditional Chinese medicine against viruses), the patient’s fever resolved, and her condition stabilized.

**Figure 2:**
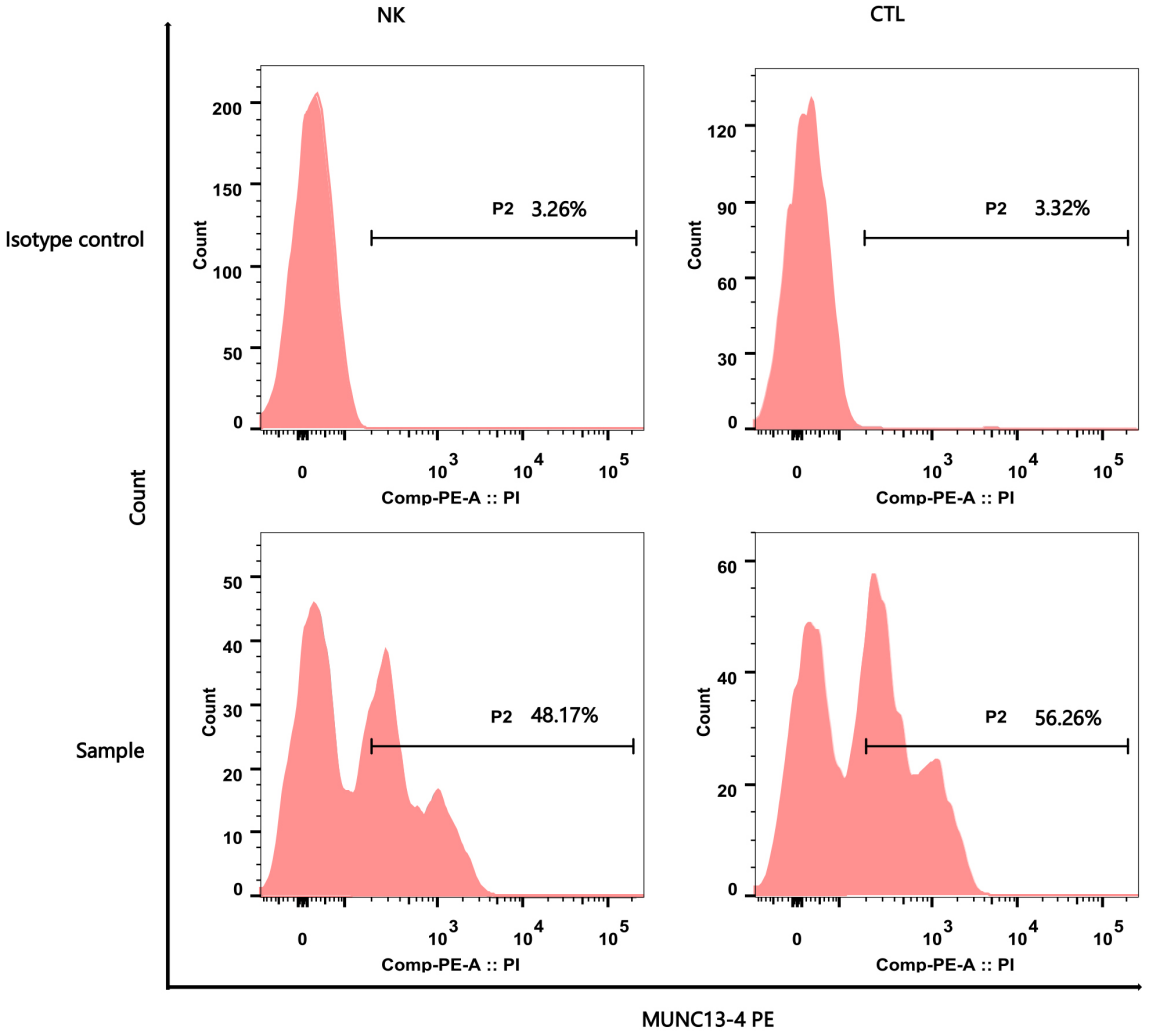
Flow Cytometry Analysis Report of MUNC13–4 (UNC13D) Expression. The upper part of the picture showed the negative marker of MUNC13–4 expression was determined by the Isotype control. The below part of the pictures show that MUNC13–4 was detected in the patient’s sample, the gate P2 represented the positive part of MUNC13–4 expression. Positive specific MUNC13–4 expression is defined as the P2 portion of the patient sample minus the isotype-labeled portion (the top half of the picture) over the Marker’s non-specific positive expression. The specific relationship is shown in **Table 1**. NK, NK cell; CTL, Cytotoxic T Lymphocyte.

The patient was admitted to our hospital in October 2018 due to the recurrent symptoms and sign mentioned above. Moderate anemia, abdominal distention, and massive splenomegaly, [Line 1 (The intersection of left midclavicular line and left costal margin to splenic lower margin): 12cm; Line 2 (The intersection of left midclavicular line and left costal margin to splenic distal point): 13cm; Line 3(The right splenic margin exceeds the Midumbilical line): +5cm], were found in her physical examination. Auxiliary examination results were as follows: blood routine test showed white blood cell count (WBC): 3.4 × 10^9^/L, neutrophil count (NE): 0.68 × 10^9^/L, hemoglobin concentration (Hb): 68g/L, platelet count (Plt): 55 × 10^9^/L, Biochemical indicators: Lactate dehydrogenase (LDH) concentration: 305U/L, albumin concentration: 32.8g/L, triglyceride concentration: 1.93mmol/L. Ferritin concentration: 1440ng/mL. Fibrinogen content (Fib):1.93 g/L, Virus series examination showed EBV-DNA level of 5.01 × 10^4^ IU/mL, Soluble CD25 > 44000 U/ml, NK cell activity of 7.45%, and MUNC13–4 (UNC13D) expression report suggested it was lower than that in normal control, which was shown in **Figure 2** and **Table 1**. A bone marrow biopsy revealed significantly active hyperplasia of nucleated cells, occasional hemophagocytic events, and immature abnormal hematopoietic elements, indicative of mixed anemia. According to the HLH-2004 diagnostic criteria (4), the patient was diagnosed with primary hemophygocytic lymphohistiocytosis. She received HLH-2004 chemotherapy in the hospital, but Initial treatment yielded unsatisfactory results. Subsequently, L-DEP regimen was used for salvage treatment (5) (pegaspargase, dexamethasone, etoposide). The standard salvage therapy for HLH is doxorubicin liposome (DOX) 25mg/M^2^ d1, etoposide (VP-16) 150mg d1, dexamethasone (DEX) 8mg/d d1-d5, pegaspargase (peg-asp) 1800U/m^2^ d3. The patient was a child with low body weight and was treated with a reduced dose of L-DEP regimen: DOX 20mg d1, VP-16 100mg d1, DEX 5mg/d d1-d5, peg-asp 1000U/m^2^ d3. After two weeks of treatment, according to the evaluation criteria proposed by Marsh et al. (6) The evaluation results showed partial remission. The patient then commenced a continuous treatment regimen of dexamethasone, etoposide, and cyclosporine A. During this period, the patient had recurrences for many times, and various rescue therapies were tried (such as L-DEP and DEP regimen), as well as discussions and considerations of HSCT. Unfortunately, the family members hesitated for a year about whether to accept HSCT.

**Table 1:** MUNC 13-4 (UNC13D) expression report.

groups	MUNC13-4 Expression Rate (%) Isotype control	MUNC13-4 Expression Rate (%) Sample	ΔMUNC13-4 (%) (Sample-ISO)	ΔMUNC13-4 (%) Normal Reference range
NK	3.26	48.17	44.91	≥51
CTL	3.32	56.26	52.94	≥60

Δ, Sample-ISO; ISO, Isotype control.

**Figure 3:**
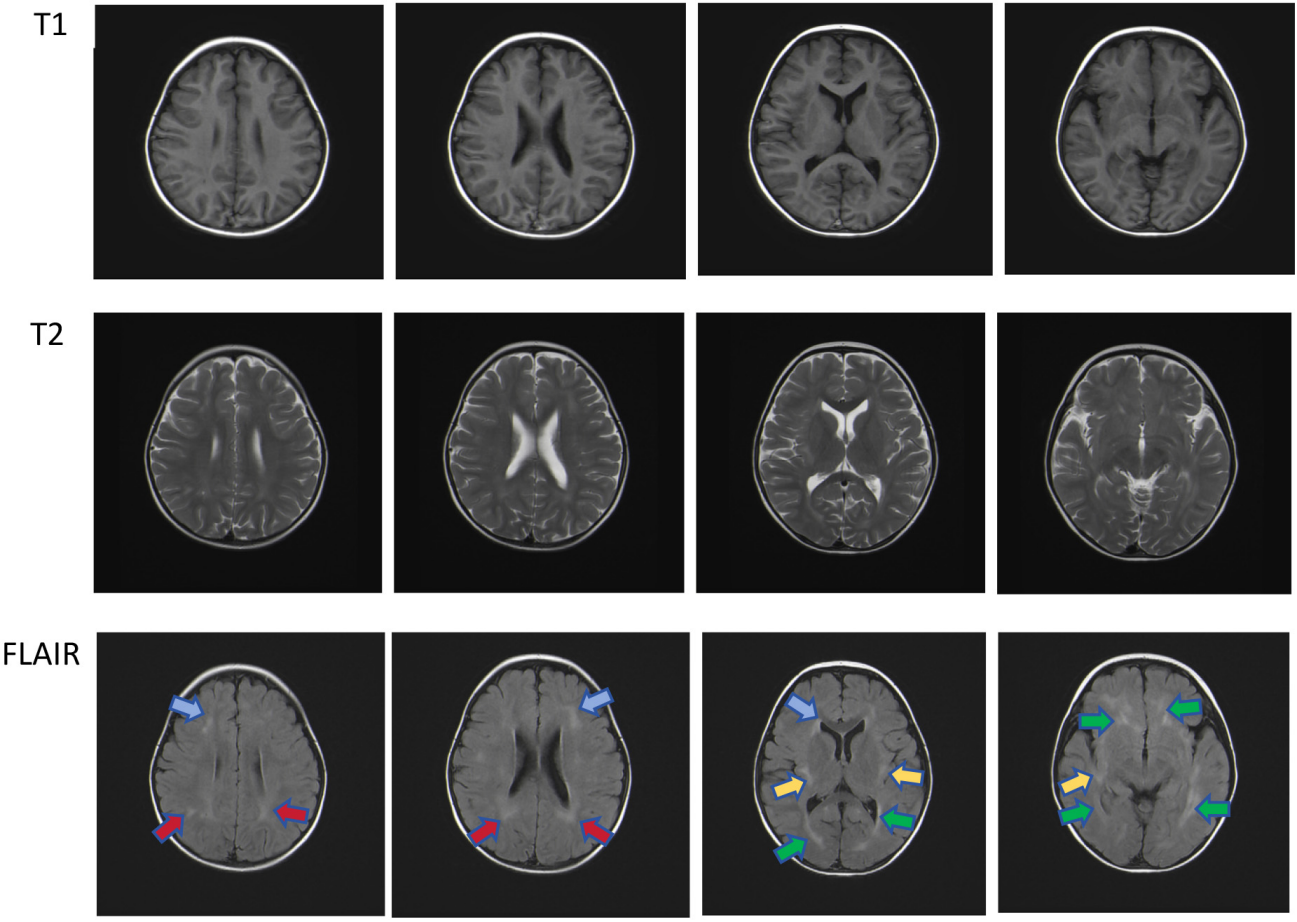
Multiple patchy T2 hyper-intensities were observed in the subcortical and deep white matter of both cerebral hemispheres, partially located around the ventricles. Similar lesions were also observed in the bilateral cerebellar hemispheres under the tentorium. Head magnetic resonance imaging shows multiple patchy hyper-intensities in the Flair images, which were located in the bilateral frontal (blue arrow), deep bilateral parietal (red arrow), sub-insular cortex (yellow arrow), and periventricular white matter (green arrow).

The patient was admitted with a primary symptom of fever persisting for three days, accompanied by seizures manifesting as involuntary twitching of the left upper limb. An allogeneic HSCT was scheduled for February 2019. Physical examination showed moderate splenomegaly [Line 1 (The intersection of left midclavicular line and left costal margin to splenic lower margin): 8.5 cm; Line 2 (The intersection of left midclavicular line and left costal margin to splenic distal point): 9 cm; Line 3(The right splenic margin exceeds the Midumbilical line): -2 cm]. The blood routine test showed pancytopenia. Lumbar puncture also was performed, but the routine, biochemical and morphological examination of cerebrospinal fluid was negative. Virus series examination showed EBV-DNA level fluctuated between 1 × 10^3^ IU/mL and 8.47 × 10^5^ IU/mL. The NK cell activity was 17.57%, and the NK-CD107a level was 3.87%, indicating degranulation defect; the CTL-CD107a (MFI) level was 1.8, also indicating degranulation defect. The SCD25 level reached 102,700 U/ml. The hemophagocytic cells with abnormal cells were easily found in bone marrow slides, and the G test and GM test were all negative. Abdominal ultrasound showed slight enhancement of intrahepatic echoes, thickening of gallbladder wall, and splenomegaly. Brain MR showed white matter damage, as depicted in **Figure 3**. Cardiac ultrasound revealed mild insufficiency of the mitral and tricuspid valves. During the treatment process, the patient received ceftriaxone sodium and tazobactam for controlling bacterial infection as well as ganciclovir for inhibiting the replication of EB virus, followed by DEP chemotherapy (DOX 20mg d1, VP-16 100mg d1, DEX 40mg/d d1-d5). Post-treatment assessments showed a reduction in spleen size and improvements in blood test results; yet the high body temperature still persisted. At present, according to FHL treatment guideline (7), the patient is deemed a suitable candidate for transplant.

### 2.2 Haploidentical allogeneic HSCT

In December 2019, the patient with familial hemophagocytic lymphohistiocytosis (FHL) persisting for over five years was unable to secure a compatible donor from the Chinese bone marrow bank. Consequently, the family requested HLA6/10 Haploidentical Allogeneic HSCT from brother to sister. The donor’s results of hereditary gene test were negative. The patient was treated with myeloablative conditioning (MAC) regimen including busulfan (0.8mg/Kg 4/d -8d∼5d), fludarabine (25mg/M^2^ 1/d -8d∼4d), cyclophosphamide (15mg/Kg 1/d -8d∼4d) and etoposide (0.07g/M^2^ 1/d, -4d). Initially, etoposide was not used due to the excessive accumulation of etoposide (3.3g/m2) in previous treatment. The conditioning began on day -8, but on day -5, the patient still had moderate fever and progressive splenomegaly with occasional seizure, which might be related to the release of inflammatory factors (IL-6: 9.44 pg/ml、IL-10: 85.74 pg/ml、IFN-γ: 28.67 pg/ml) caused by FHL (**Table 2**). Considering this situation, the conditioning regimen was adjusted to add methylprednisolone 200mg (1/d, -5d∼ -3d) to control hemophagocytic syndrome. On day -4, the patient still had fever and splenomegaly without significant improvement, 0.07g etoposide and 2.5mg BID ruxolitinib (-4d ∼-2d) was added to conditioning regimen to clear inflammatory factors produced by hemophagocytic cells. After removing hemophagocytic cells with busulfan and cyclophosphamide, some cytokines (IL-5、IL-6、IL-1β、IL-8) that promote inflammatory responses were released in blood, so plasma exchange was added on day -2 to reduce these cytokine levels. After plasma exchange, the patient’s consciousness, symptoms, body temperature and spleen size (Line 1: 6cm; Line2: 7cm, Line 3: -1cm) were improved, and no neurological symptoms occurred. Eventually donor peripheral blood stem cells (PBSC) were reinfused: NC 93.92×10^8^/kg, MNC 33.80×10^8^/kg, and CD34 12.48 ×10^6^/kg. Given the high risk of EBV infection (6.02 × 10^4^ IU/mL) before transplant, the slow speed of immune reconstitution, high recurrent EBV infection rate, and increased incidence of post-transplant lympho-proliferation-related disease (PTLD), post-transplant cyclophosphamide (PT-Cy) was given on +3d and +4d. On the other hand, the patient experienced reactive disease before a salvage transplant which was high probability of relapse (8). To improve patient survival, transplant strategies need to be optimized. Existing literature reported higher doses of CD34 positive cells can improve poor engraftment (PGF), but may also increase the risk of GVHD. Timothy et al. (9) showed that higher CD34 cell doses (>7.5×10^6^/kg) were associated with better 5-year OS and improved engraftment, but with an increased risk of chronic GVHD. Besides, Shiratori et al. (10) suggested that low-dose ATG (2mg/kg) could reduce severe acute and chronic GVHD after myeloablative conditioning in adults. ATG given pre-transplant has a long half-life, and it lasts until about 1 month after transplantation (11). From these literatures, we can infer that there is also a certain concentration of ATG within 1 month after transplantation. Prior to transplantation, this patient had relapsed FHL due to recurrent EBV infection. Stable and rapid functional recovery of NK cells after transplantation is the key to reduce recurrence. Thus, the strategy of increasing donor NK cell infusion is expected to reduce FHL recurrence without increasing GVHD. In this patient’s transplantation, we selected high MNC infusion volume, applied PT-Cy and low dose ATG to remove T cells, and retained the number of NK cells, in order to achieve this transplantation strategy optimization, which was also confirmed by literature. A retrospective study shows: ATG+PT-Cy developed the quicker reconstitution in some NK cell subtype which may help with avoiding relapse in haploidentical transplant (12, 13).

**Table 2:** Cytokines of the patient.

Time (-/+)	IL-5 (pg/ml)	IFN-α (pg/ml)	IL-2 (pg/ml)	IL-6 (pg/ml)	IL-1β (pg/ml)	IL-10 (pg/ml)	IFN-γ (pg/ml)	IL-8 (pg/ml)	IL-17 (pg/ml)	IL-4 (pg/ml)	IL-12p70 (pg/ml)	TNF-α (pg/ml)
-5d	N	N	N	9.44↑	N	85.74↑	28.67↑	N	N	N	N	N
+7d	13.86↑	N	N	7.60↑	29.29↑	N	N	34.91↑	N	N	N	N
+17d	N	N	N	7.39↑	19.53↑	N	N	26.06↑	N	N	N	N

-, pre-transplant; +, after-transplant; N, normal; ↑, Above normal; d, Day.

The patient was a child with a low body weight and was given a low-dose ATG regimen because the spleen that did not shrink to normal would withhold a portion of the implanted cells during cell transfusion. Hence, 17mg ATG (1mg/kg) was given on +5d to reduce acute GVHD, while the recovery of NK cells was not affected. On the +24th day after transplant (1st stem cell infusion), the bone marrow aspiration showed abnormal nuclear morphology and phagocytosis signs of erythroid hematopoietic components; meanwhile, chimerism analysis on day +26 showed that the T cell chimerism was full donor chimerism (98.2%), and the granulocyte (92%) and B cell (90%) were mixed chimerism. While on the +28d after transplant (1st stem cell infusion), the blood routine test showed WBC 0.7×10^9^/L, NE 0.3×10^9^/L, Hb 77g/L, and Plt 36×10^9^/L (after platelet and red blood cells infusion); which suggested that PGF may be present despite the shrunk spleen size (Line 1: 2cm; Line2: 3cm, Line 3: None). Thus, preserved donor PBSC were reinfused again and without conditioning regimen for just a cell boost given: NC 18.494×10^8^/kg, MNC 4.82×10^8^/kg, and CD34 2.12 ×10^6^/kg on the 28th day after transplant (1st stem cell infusion); In addition, hetrombopag olamine were also used to promote platelet production. The granulocyte engraftment was at 29th days after transplant, and the platelet engraftment at +48 days after transplant, and it took about 110 days for the platelets return to normal. Chimerism analysis was performed on day +50: T cell chimerism was full donor chimerism (99.8%), granulocytes (99.75%) and B cells (98.21%) were full chimerism (all above were calculated on the first day of cell infusion). The patient’s temperature remained normal, and the spleen size reduced to 3cm sub-costally by day +50. EBV status converted to negative within a week post-transplant. All of these symptoms suggested that the patient’s FHL had been well controlled. After the condition was stabilized, the patient was discharged from the hospital and was followed up regularly at the outpatient department.

## 3 Literature search

Hemophagocytic Lymphohistiocytosis (HLH) is a rare disorder characterized by excessive immune activation and uncontrolled inflammation. In China, the total incidence of HLH was reported to be approximately 1.04‰ in 2019 (14). HLH can be divided into primary HLH and secondary HLH. Primary HLH, including FHL and immune deficiency syndrome-associated HLH, is an autosomal recessive genetic disease that mostly occurs in infants. The genes mutations known as related to primary HLH include PRF1, UNC13D, STXI11, STXBP2, LY-ST, RAB27A, ADTB3A, SH2D1A, XIAP, BIRC4, ITK, CD27 and MAGT1 (1). Viral infections, especially EB virus, are the most common infection in patients with HLH (15). A retrospective study (16) has highlighted that primary HLH patients with central nervous system involvement tend to have a worse prognosis. The 3-year overall survival of patients with central nervous system involvement is significantly lower than that of patients without central nervous system involvement. Early recognition and prompt treatment are crucial for improving outcomes in HLH cases.

Due to the lack of specific clinical manifestations of HLH, misdiagnosis is easily caused. The severity of its clinical manifestations is related to the degree of immune cell activation and cytokine levels (17). The HLH-2004 diagnostic criteria, updated by the International Society of Histology and Cell Biology in 2004, are the benchmark for diagnosing HLH (2). According to these criteria, a diagnosis can be confirmed if any of the following two criteria are met: (1) molecular genetic test results are consistent with HLH-related pathogenic gene mutations, such as PRF1, UNC13D, STXI11, STXBP2, etc.; (2) at least five of the following eight indicators are met simultaneously: (1) persistent body temperature >38.5°C for more than 7 days; (2) splenomegaly; (3) significant bilineage or trilineage cytopenia in peripheral blood: hemoglobin <100 g/L in infants <4 weeks of age, or <90 g/L in other cases; platelet count <100×10^9^/L; neutrophil count <1.0×10^9^/L not caused by bone marrow hypoplasia; (4) Increased triglyceride (TG) and/or decreased fibrinogen: TG >3 mmol/L or more than 3 standard deviations above the age group, fibrinogen <1.5 g/L or less than 3 standard deviations above the age group; (5) Hemophagocytosis was observed in the bone marrow, spleen, liver or lymph nodes; (6) Reduced or absent NK cell activity; (7) Increased ferritin level: ferritin ≥500 ng/mL; (8) Increased sCD25 (soluble IL-2 receptor).

EB virus-associated hemophagocytic lymphohistiocytosis (EBV-HLH) is a common subtype of HLH in Asian countries. In EBV-HLH patients, infected T cells overproduce inflammatory cytokines, leading to macrophage activation. The present case involves a pediatric patient who developed familial HLH (FLH) as a result of recurrent EBV infections, with the added complexity of central nervous system involvement. After a challenging course, the child ultimately underwent successful salvage transplant.

A study has shown (16) that 40%-70% of pediatric HLH patients show central nervous system involvement. HLH with central nervous system involvement is often characterized by nonspecific neurological symptoms and signs, such as seizures, mental status changes, pseudo-meningitis, and focal neurological signs. In the case presented, the patient experienced transient seizures, highlighting the clinical challenge posed by CNS-HLH. Currently, there is an absence of definitive diagnostic criteria for CNS-HLH, making its assessment reliant on a combination of neurological signs/symptoms, neuroimaging abnormalities, and cerebrospinal fluid (CSF). The preferred modality is MRI of the brain. MRI imaging features are highly nonspecific and variable, such as multiple white matter lesions (66%), cerebellar encephalitis (19%), and brainstem dominant disease (15%) (18). In pediatric patients, common radiological signs include periventricular white matter hyper-intensity and generalized brain atrophy (19). Moreover, a significant number of individuals with CNS-HLH present with CSF abnormalities. These can manifest as increased cellularity, pleocytosis, elevated protein concentration, and the presence of hemophagocytosis (20).

Early diagnosis and identification of risk factors is crucial in managing secondary hemophagocytic lymphohistiocytosis (sHLH). In a retrospective study of 162 patients with sHLH (21), a minimal parameter set was found which consisting of 2 major criteria (hemophagocytosis and splenomegaly) and 3 minor criteria (cytopenia, increased ferritin, and increased triglycerides/low fibrinogen). HLH was most likely when a patient either had 2 major positive criteria, 1 major and 2 minor positive criteria, or 3 minor positive criteria. In a study of Saralee et al. (22), central nervous system involvement and low baseline platelet count are independent predictors of early death in children with HLH. Meanwhile, Zhou et al. (23)showed that if albumin <25 g/L, APTT >65s, LDH >1000 U/L, or age <28 months at diagnosis were independent risk factors for poor early prognosis in children with HLH. These findings emphasize the importance of early detection and risk assessment in managing sHLH.

Once diagnosed with HLH, it is imperative to initiate systemic treatment with immunosuppressants or anti-inflammatory drugs promptly, alongside targeted therapies for associated infections, pancytopenia, and coagulation dysfunction, to halt disease progression. Allogeneic HSCT is considered the only curative therapy (24). The International Association of Tissue Cells found in numerous clinical studies that the 5-year survival rate of FHL patients receiving HSCT was 50%, while all children who did not receive HSCT died (25). For FHL, those patients as the candidates for HSCT will have the greatest chance of being cured. A retrospective study on pediatric HLH (26)showed that the patients based on the time interval from diagnosis to transplant were divided into a short-time interval group and a long-time interval group, and the 3-year OS rates of the two groups were 83.3% and 66.7%, respectively. Even if there is active disease at the time of transplant, HSCT is an important treatment for CNS-HLH. And patients with FLH may also benefit from immediate HSCT (27). Therefore, we inferred that the prognosis of early transplant following partial response yields a better prognosis than delayed transplant following complete remission in children with hemophagocytic syndrome. At the same time, Greental et al. (28) showed that 5-year EFS rates were higher with MAC regimen for transplant from either HLA matched or alternative donors, so the patient used MAC as conditioning regimen. Certainly controlling hemophagy was also a vital factor for achieving long-term survival. This case is a HLA6/10 Haploidentical Allogeneic HSCT from brother to sister with MAC regimen. Before transplant, factors such as disease activity and splenomegaly can affect the engraftment of donor cells. If hemophagy is not controlled, poor retraction of the spleen often results in PGF, as exemplified in this case. However, there is no standard conditioning regimen before transplant. Studies have shown that plasma exchange can control the release of cytokines related to HLH, and is critical means before transplant in patients who fail to respond to traditional treatment (29, 30). Additionally, etoposide and busulfan also play a crucial role in removing hemophagocytic cells (7, 31, 32). Etoposide was not included in the original conditioning regimen for this patient, while the hemophagy was not controlled, so we had to introduce etoposide and plasma exchange into conditioning regimen to control the hemophagy. Therefore, we believe that the future optimization of FHL conditioning regimen should pay attention to retaining etoposide and adding plasma exchange at an appropriate time, which can enhance the control of the disease, but does not affect the effects of other drugs. In this case, despite an increased CD34+ cell dose, this patient required a subsequent infusion to achieve stable engraftment. Inadequate disease control at the time of salvage HSCT is a significant contributor to engraftment failure. Prioritizing this aspect can diminish the risk of PGF.

Overall, this study reports a case of FHL caused by UNC13D mutation treated with allogeneic HSCT, providing valuable clinical experience in salvage therapy of FHL. Given that HSCT is currently the ultimate curative treatment for FHL, and in order to make HLH patients get a bright prognosis, more optimized conditioning regimens, higher engraftment rate, and better control of inflammatory cytokine storm occurring during transplant are still the important directions in the future.

## Data availability statement

The raw data supporting the conclusions of this article will be made available by the authors, without undue reservation.

## Ethics statement

Written informed consent was obtained from the individual(s), and minor(s)’ legal guardian/next of kin, for the publication of any potentially identifiable images or data included in this article.

## Author contributions

BL: Conceptualization, Data curation, Formal analysis, Investigation, Methodology, Software, Writing – original draft, Writing – review & editing. LLZ: Data curation, Formal analysis, Resources, Supervision, Writing – review & editing, Investigation, Methodology. FM: Data curation, Investigation, Methodology, Supervision, Writing – review & editing. XF: Investigation, Methodology, Supervision, Writing – review & editing. DH: Supervision, Writing – review & editing, Investigation, Methodology. XW: Supervision, Writing – review & editing. LXZ: Data curation, Software, Supervision, Writing – review & editing. FW: Supervision, Writing – review & editing. XZ: Methodology, Supervision, Writing – review & editing. WY: Conceptualization, Data curation, Formal analysis, Funding acquisition, Investigation, Methodology, Project administration, Resources, Software, Supervision, Validation, Visualization, Writing – original draft, Writing – review & editing.
